# Effect of HIF-1α inhibitor LW6 on chondrogenic properties of mesenchymal stromal cells and chondrocytes in cell sheets under physioxia

**DOI:** 10.1186/s13036-025-00588-8

**Published:** 2026-01-09

**Authors:** Ignas Lebedis, Jolita Pachaleva, Eiva Bernotiene, Daiva Bironaite, Tomas Ragauskas, Giedrius Kvedaras, Gunaras Terbetas, Ilona Uzieliene

**Affiliations:** 1 Department of Regenerative Medicine, State Research Institute Centre for Innovative Medicine, Santariskiu str. 5, Vilnius, LT-08406 Lithuania; 2 Faculty of Fundamental Sciences, Vilnius Tech, Sauletekio al. 11, Vilnius, LT-10223 Lithuania; 3 The Clinic of Rheumatology, Orthopaedics Traumatology and Reconstructive Surgery, Faculty of Medicine, Vilnius University, M. K. Ciurlionio str. 21, Vilnius, LT-03101 Lithuania; 4 The Clinic of Neurology and Neurosurgery, Faculty of Medicine, Vilnius University, M. K. Ciurlionio str. 21, Vilnius, LT-03101 Lithuania

**Keywords:** Mesenchymal stromal cells, Chondrocytes, Chondrogenic differentiation, LW6, Physioxia, Cell sheet technology

## Abstract

**Background:**

Hypoxia-inducible factor 1 (HIF-1) plays a central role in cartilage homeostasis, and its modulation may influence the performance of cell-based therapies. LW6, an inhibitor of HIF-1α and a potential anti-cancer therapeutic, has not been previously evaluated in cartilage regeneration-related studies and chondrogenic cell sheet systems. The aim of this study was to investigate how LW6 affects the intracellular functions and chondrogenic potential of human bone marrow–derived mesenchymal stromal cells (BMMSCs) and chondrocytes cultured in 2D, and as scaffold-free cell sheets under physioxic (5% O₂) or normoxic (21% O₂) conditions.

**Results:**

Physioxia enhanced proliferation, suppressed mitochondrial and malate dehydrogenase 2 activity, reduced glycolysis, lowered intracellular calcium but stabilized HIF-1α and increased the expression of chondrogenic genes (SOX9, COL2A1, ACAN) in both cell types. LW6 further reduced metabolic activity and calcium levels, degraded HIF-1α and downregulated chondrogenic gene expression, particularly in chondrocytes. Physioxia also improved cell sheet integration with human cartilage explants and ECM deposition.

**Conclusions:**

Physioxic conditions support cartilage-forming potential and improve biointegration of BMMSC and chondrocyte cell sheets with human cartilage explants, while HIF-1α modulator LW6 negatively affects chondrogenic and metabolic pathways. These findings provide new insights into how HIF-1-targeting compounds may influence cell–based cartilage engineering and highlight the need for careful consideration of HIF-1 modulation in regenerative applications.

## Background

Human articular cartilage has a limited self-regenerative capacity after being damaged, making it vulnerable to progressive degeneration and diseases such as osteoarthritis (OA). Although research on cartilage regeneration in vitro is promising, no fully effective treatments for cartilage lesions yet exist. Mesenchymal stromal cells (MSCs) have shown potential in cartilage repair due to their ability to differentiate into chondrocytes and stimulate tissue regeneration [1]. However, applications of MSCs in vivo remain largely insufficient due to challenges such as immunogenicity, difficulty of transplantation, biointegration and issues related to tissue scaffolds [2, 3]. Therefore, it is necessary to explore new cartilage regeneration strategies using scaffold-free technologies.

Cell sheet technology is an innovative approach that utilizes a patient’s autologous cells to promote the repair of damaged tissues [4–6]. Transplanting these intact cell sheets directly onto degraded tissue has been proven to show effective biointegration and easier engraftment to host tissue, while promoting cell signaling during regeneration compared to conventional cell therapy treatments. An important aspect of chondrogenesis in the human body is the naturally occurring hypoxic (physioxic) environment of cartilage tissue, ranging from 5% to less than 1% O_2_ [7]. The most important regulators of cell response to hypoxic conditions are hypoxia-inducible factors (HIFs). HIFs are transcription factors known to promote cell survival in the absence of oxygen, and in the case of cartilage regeneration HIF-1 plays a significant role in differentiation and gene transcription of cells related to the formation, upkeep of cartilage tissue and its surrounding extracellular matrix (ECM) [8, 9].

LW6, or 3-[2-(4-Adamantan-1-yl-phenoxy)-acetylamino]-4-hydroxy-benzoic acid methyl ester, is a relatively novel synthetic compound and HIF-1α inhibitor, which doesn’t directly inhibit HIF-1α, but lowers its protein accumulation and thus HIF-1 protein expression in cells without affecting HIF-1β [10]. It was shown in previous studies that LW6 induced apoptosis in a hypoxic human lung cancer cell line through depolarization of mitochondria [11], and demonstrated anti-tumor efficacy in mice via decreasing HIF-1α expression through the Von Hippel-Lindau protein (VHL)-dependent proteasome system pathway [12]. VHL is involved in the ubiquitination and degradation of HIF-1α, a subunit of HIF-1 that plays a central role in regulating gene expression in response to oxygen levels. Another mode of LW6 action was shown by the inhibition of the activity of malate dehydrogenase 2 (MDH2) [13, 14]. As a mitochondrial protein, MDH2 is a key enzyme in the tricarboxylic acid (TCA) cycle that catalyzes the interconversion of malate and oxaloacetate by utilizing the NAD/NADH coenzyme system [15].

Although LW6 has been proposed as a promising anti-cancer compound due to its ability to suppress HIF-1α accumulation, its broader biological effects on non-cancerous, HIF-dependent tissues remain largely unknown. Because HIF-1α is essential for chondrogenesis and cartilage homeostasis, any off-target inhibition could adversely affect cell metabolism, differentiation, or matrix formation. Therefore, the purpose of this study was to investigate whether LW6 induces unintended, potentially deleterious effects on bone marrow MSCs (BMMSCs) and chondrocytes, particularly under physioxic conditions that resemble the native cartilage environment. By analysing intracellular responses, metabolic activity, and chondrogenic differentiation in 2D culture and cell-sheet models, we aim to clarify the safety considerations of LW6 for applications where HIF-1 signaling is physiologically important. Given the essential role of HIF-1α in cartilage development and homeostasis, we hypothesized that LW6 may negatively affect BMMSC and chondrocyte function by disrupting HIF-dependent metabolic and chondrogenic pathways. **Thus**,** the aim of this study was to evaluate intracellular responses and chondrogenic potential of human BMMSCs and chondrocytes grown in chondrogenic cell sheets and treated with the novel HIF-1α inhibitor LW6 under physioxic (5% O**_**2**_**) and normoxic (21% O**_**2**_**) conditions.** We also hypothesize that physioxic conditions better stimulate chondrogenic potential compared with normoxia, while the HIF-1α modulator/inhibitor LW6 suppresses it.

## Materials and methods

### Cell isolation and culture

BMMSCs and chondrocytes were isolated and cultivated as previously described [16]. Post-surgical bone marrow and cartilage samples were collected at Santaros hospital (Vilnius, Lithuania). All procedures using human donor tissues in this study were performed in compliance with the Bioethical Permission (No. 158200-14-741-257), approved by the Vilnius Regional Biomedical Research Ethics Committee. All donors signed informed consent forms for donating their tissues.

The bone marrow, received from 28 to 32 year old females (*n* = 5), was gradiently-centrifuged and filtered into a sterile tube, while chondrocytes were isolated from 40 to 82 year old female knee OA cartilage samples (*n* = 5) using enzymatic digestion by pronase (Roche diagnostics, Basel, Switzerland), followed by type II collagenase (Biochrom AG, Berlin, Germany). All cell types were seeded into cell culture flasks for further cultivation.

BMMSCs and chondrocytes were cultivated under the same conditions in complete growth medium – low glucose DMEM medium (Thermo Fisher Scientific, Waltham, MA, USA) supplemented with 10% fetal bovine serum FBS (Thermo Fisher Scientific, Waltham, MA, USA) and 1% PS (penicillin 10,000 units/mL and streptomycin 10,000 µg/mL, Sigma Aldrich, St. Louis, MO, USA) in a cell culture incubator with 37 °C and 5% CO_2_. Cells were re-seeded or used for experiments when reaching 80% confluence. Early passages (up to *p* = 5) of BMMSCs and chondrocytes were used in all experiments, with medium changes twice a week.

For normoxic (37 °C, 21% O_2_ and 5% CO_2_) and physioxic (37 °C, 5% O_2_ and 5% CO_2_) conditions during experiments, cells were cultivated in a standard cell culture incubator and hypoxia chamber Xvivo System X3 (Biospherix, Parish, NY, USA), respectively.

#### Cell proliferation/metabolic assay

For the cell proliferation analysis, BMMSCs and chondrocytes were seeded into 12 well plates (20,000 cells/well) and cultured in complete growth medium supplemented with 10 µmol LW6 under normoxic and physioxic conditions, changing the medium three times a week. For controls, wells containing only complete medium were used. After 3 and 7 days, cells were washed with PBS, medium with 4% WST-8 reagent from the Cell Counting Kit 8 (CCK-8) (Dojindo Laboratories, Kumamoto, Japan) was added, and the cells incubated under normoxic and physioxic conditions for 3 h. The CCK-8 kit, consisting of a tetrazolium salt, allows analysis of cellular metabolic activity/cellular proliferation by dehydrogenase activity and reduction of tetrazolium to a formazan dye. After incubation, the medium was collected and its optical density was measured at 450 nm with a microplate reader SpectraMax i3 (Molecular Devices, San Jose, CA, USA). LW6 was used at 10 µM for all following studies, a concentration selected based on prior studies demonstrating effective HIF-1α/MDH2 inhibition without cytotoxicity [12–14].

#### Cell viability assay

The viability of BMMSCs and chondrocytes with HIF-1α inhibitor LW6 was analyzed using the LIVE/DEAD™ Viability/Cytotoxicity Kit (Thermo Fisher Scientific, Waltham, MA, USA). Cells were seeded in 12 well plates (20,000 cells/well) and cultured in complete growth medium supplemented with 10 µmol LW6 (Santa Cruz Biotechnology, Dallas, TX, USA) for 21 days under normoxic and physioxic conditions, changing the medium two times a week. For controls, wells containing only complete medium were used. After 21 days, the cells were washed twice with phosphate buffered saline (PBS) (Sigma Aldrich, St. Louis, MO, USA) and the kit reagents (calcein-AM and ethidium homodimer-1) were added. Staining and incubating the cells under normoxic and physioxic conditions was done according to the manufacturer’s instructions. Cell viability was analyzed by fluorescent microscope EVOS M7000 Imaging System (Thermo Fisher Scientific, Waltham, MA, USA) according to the recommended excitation/emission wavelengths.

#### Lactate dehydrogenase (LDH) assay

BMMSCs and chondrocytes were seeded into 12 well plates (20,000 cells/well) and cultured in complete growth medium supplemented with 10 µmol LW6 in normoxic and physioxic conditions for 21 days. For controls, wells containing only complete medium were used. Afterwards, the cell medium was collected into 96 well plates and analyzed with the CyQUANT LDH Cytotoxicity Assay kit (Thermo Fisher Scientific, Waltham, MA, USA) according to the manufacturer’s protocol with an included positive LDH control. The optical density was measured with a microplate reader SpectraMax i3.

#### Intracellular calcium ion assay using flow cytometry

BMMSC and chondrocytes were seeded into 6-well plates (50,000 cells/well) and cultured in complete growth medium supplemented with 10 µmol of LW6 in normoxic and physioxic conditions for 3 and 7 days. For controls, wells containing only complete medium were used. After the 3rd and 7th day of cultivation, cells were detached with 0.25% trypsin-EDTA (Thermo Fisher Scientific, Waltham, MA, USA), collecting 50,000 cells of each group into flow cytometry tubes, centrifuged at 500× g for 5 min, stained with 1 µM of calcium specific fluorescent dye Cal-520 (Santa Cruz, Biotechnologies, Dallas, TX, USA) and incubated in complete growth medium under normoxic and physioxic conditions for 30 min. After incubation, cells were washed with PBS and centrifuged at 500× g for 5 min. The supernatant was discarded, and cells were resuspended in 300 µL of PBS. Each sample was run in triplicates; fluorescence was measured using a flow cytometer Guava (Cytek, Fremont, CA, USA), 488 nm laser paired with a 530/30 nm bandpass filter. The data was analyzed using FlowJo software version 10 (FlowJo LLC, Ashland, OR, USA). The median fluorescence intensity (MFI) of all samples was calculated by subtracting the MFI of non-stained cells from the MFI of Cal-520 stained cells.

#### Cell respiration assays

The mitochondrial respiration and glycolysis (oxygen consumption rate (OCR) and extracellular acidification rate (ECAR)) of BMMSC and chondrocytes was measured and analyzed using the Seahorse XFp Cell Mito Stress Test and Seahorse XFp Cell Glycolysis Stress Test kits (Agilent Technologies, Santa Clara, CA, USA). Cells were seeded into Seahorse XFp FluxPak plates (Agilent Technologies, Santa Clara, CA, USA) (5,000 cells/well) and cultivated under normoxic and physioxic conditions for 3 days, supplementing the medium with 10 µmol LW6. For controls, wells containing only complete medium were used. After 3 days, the plate sensor cartridges were prepared as described by the manufacturer protocol, added to the cell plates and inserted into a Seahorse XF HS Mini metabolic analyzer (Agilent Technologies, Santa Clara, CA, USA). Metabolic analysis was performed according to the in-system provided protocols for the supplied kits.

#### ELISA for HIF-1α and VHL proteins

For the analysis of hypoxia and LW6 associated proteins, BMMSCs and chondrocytes were seeded into 6 well culture plates (50,000 cells/well) and cultivated under normoxic and physioxic conditions for 3 and 7 days, supplementing the medium with 10 µmol LW6. For controls, wells containing only complete medium were used. For Hypoxia-inducible factor alpha (HIF-1α) analysis, the PathScan Total HIF-1α Sandwich ELISA Kit was used (Cell Signaling Technology, Danvers, MA, USA). Cells were washed with PBS, incubated on ice with Cell Lysis Buffer (1X) supplemented with Protease/Phosphatase Inhibitor Cocktail (1X) (Cell Signaling Technology, Danvers, MA, USA) for 5 min, then scraped off, centrifuged in a 5415R centrifuge (Eppendorf, Hamburg, Germany) for 10 min (16,100 × g at 4 °C) and stored at -80 °C. The test procedure was performed according to the kit protocol, incubating the plates with cell lysate for 2 h at 37 °C. The sample OD was measured spectrophotometrically at 450 nm with a microplate reader SpectraMax i3.

For VHL protein analysis, the Human Von Hippel-Lindau Tumor Suppressor (VHL) ELISA Kit (Abbexa, Cambridge, UK) was used. Cells were collected by washing them three times with PBS, detaching with 0.25% trypsin-EDTA, suspending in PBS and freezing/thawing them three times at -20 °C. Cells were then centrifuged for 10 min (1500 × g at 4 °C) and the supernatant was collected for analysis. Reagent preparation and assay protocol steps were performed according to the kit provided protocol, incubating the samples with TMB substrate for 30 min before adding the Stop Solution. The sample OD was measured spectrophotometrically at 450 nm with a microplate reader SpectraMax i3, and concentration calculated by standard curve.

#### MDH2 activity assay

To check the inhibitor activity of LW6, an assay of MDH2 activity was performed using the Mitochondrial MDH2 Activity Assay Kit (Abcam, Cambridge, UK). BMMSCs and chondrocytes were seeded into 6 well culture plates (50,000 cells/well) and cultivated under normoxic and physioxic conditions for 3 and 7 days, medium supplemented with 10 µmol LW6. Cells were washed twice with PBS and scraped, while solubilizing them in the provided extraction buffer, supplemented with Protease/Phosphatase Inhibitor Cocktail (1X) (Cell Signaling Technology, Danvers, MA, USA). Reagent preparation and the assay procedure were performed according to the provided kit protocol, measuring the sample OD at 450 nm after 30 min with a microplate reader SpectraMax i3.

#### Chondrogenic differentiation in cell sheets

BMMSCs and chondrocytes were seeded into 12 well temperature-responsive cell sheet culture plates (Nunc™ UpCell™, Thermo Fisher Scientific, Waltham, MA, USA) (200,000 cells/well) then cultured in complete growth medium at 37 °C and 5% O_2_. After the cells reached confluence, complete growth medium was replaced with chondrogenic medium containing high glucose DMEM (Thermo Fisher Scientific, Waltham, MA, USA), 1% PS, 1% Insulin-Transferrin-Selenium-Ethanolamine (ITS-X) (Thermo Fisher Scientific, Waltham, MA, USA), 0.35 mM L-proline (Carl Roth, Karlsruhe, Germany), 10⁻⁷ M dexamethasone (Sigma Aldrich, St. Louis, MO, USA), 0.17 mM ascorbic acid phosphate (Sigma Aldrich, St. Louis, MO, USA) with 10 ng/mL of growth factor TGF-β3 (Thermo Fisher Scientific, Waltham, MA, USA). Cells were placed into normoxic or physioxic conditions and chondrogenic differentiation in cell sheets was stimulated for 21 days, changing the medium three times a week. Afterwards, the cell sheets were detached from the temperature-responsive surface. For this purpose plates were moved to room temperature (approximately 20–25 °C) for 30–60 min, which lowers the culture surface hydrophobicity and allows non-enzymatic release of confluent sheets. Sheets were released by gently pipetting room-temperature medium along the well edges using a wide-bore pipette tip (or by gently tilting the plate). No trypsin or mechanical scraping was used, preserving the ECM and cell to cell contact. Detached sheets were used for further experiments – RT-qPCR, or cartilage biointegration study.

#### RNA extraction from cell sheets

After 21 days of chondrogenic differentiation, cell sheets were detached from culture plates, washed twice with PBS and collected into microtubes, storing them at -70 °C in RLT buffer from the RNeasy Mini Kit (Qiagen, Hilden, Germany) supplemented with 10% 2-mercaptoethanol (Carl Roth, Karlsruhe, Germany). RNA was subsequently extracted and purified by RNeasy Mini Spin columns according to the manufacturer’s protocol. The concentration and purity of RNA were measured by spectrophotometry, storing the samples at -20 °C afterwards.

#### cDNA synthesis and RT-qPCR

After obtaining RNA samples, dsDNase treatment and cDNA synthesis were performed with the Maxima First Strand cDNA Synthesis Kit (Thermo Fisher Scientific, Waltham, MA, USA) according to the manufacturer’s protocol. RT-qPCR was performed by preparing the samples in technical triplicates using Maxima Probe qPCR Master Mix (2X) (Thermo Fisher Scientific, Waltham, MA, USA) with the QuantStudio 1 Real-Time PCR System (Thermo Fisher Scientific, Waltham, MA, USA). TaqMan Real-Time Gene Expression Assays (Thermo Fisher Scientific, Waltham, MA, USA) were used for gene expression analysis, using the genes SOX9 (assay ID Hs00165814_m1), COL2A1 (assay ID Hs01060345_m1) and ACAN (assay ID Hs00153936_m1). Cycle conditions were as follows: initial denaturation step at 95 °C for 10 min, followed by 40 cycles of denaturation at 95 °C for 15 s and finally annealing and extension at 60 °C for 60 s. Controls were used for each RNA sample to check genomic DNA contamination (no reverse transcriptase control) and reagent contamination (no template control). Two reference genes – RPS9 (assay Hs02339424_g1) and B2M (assay Hs00984230_m1) were used for normalization of gene expression.

Relative levels of gene transcripts were calculated by subtracting the threshold cycle (Ct) of the geometric mean of the two reference genes from the Ct of the gene of interest, giving dCt values that were subsequently transformed to 2−ᵈᶜᵗ values and multiplied by 1000 to scale-up for better graphical representation.

#### Cell sheet biointegration to cartilage explants

Cell sheet biointegration studies were performed by first obtaining human articular cartilage from OA patients undergoing surgery in Santaros Hospital. The obtained cartilage was washed with PBS containing 2% PS and transferred to a large Petri dish. The cartilage was cut into flat, rectangular explants of similar height, width and surface area. The cartilage explants were washed and transferred to a 37 °C incubator with 5% CO_2_ for 24 h, culturing them in high glucose DMEM with 2% PS. The next day, cartilage explants were transferred to 12 well plates with BMMSC or chondrocyte cell sheets placed on the surface. Explant cultures and cell sheets were left to attach for 7 days under normoxic or physioxic conditions, changing the medium thrice a week. Biointegration of explants and cell sheets was observed and quantified by histological analysis.

#### Histology assay

For the histological analysis of cartilage explants and cell sheets, the samples were collected and fixed in 10% neutral buffered formalin solution (Sigma Aldrich, St. Louis, MO, USA), then embedded into paraffin wax and cut by microtome into 4-micrometer sections. The sections were deparaffinized and stained with safranin-O (Thermo Fisher Scientific, Waltham, MA, USA). Stained sections were analyzed by light microscopy.

#### Statistical analysis

The main statistical difference between groups was evaluated using two-way ANOVA with Tukey’s multiple comparison tests applied for group analysis and statistical significance at *p* ≤ 0.05. GraphPad Prism 9.0.0 software was used to perform statistical analysis. Three to five patients’ cells and three technical repeats were measured.

Statistical difference for intracellular calcium levels was calculated by unpaired t-test and statistical significance at *p* ≤ 0.05.

Statistical differences of gene expression were calculated as ratios: (1) normoxia vs. physioxia; (2) normoxia with LW6 vs. control (normoxia untreated); (3) physioxia with LW6 vs. control (physioxia untreated). Other statistical two-way ANOVA calculations were done comparing effects between the cell physiological conditions and cells treated vs. untreated with LW6. The exact group analysis is provided in each Figure’s legends.

## Results

### Proliferation/metabolic activity and viability of BMMSCs and chondrocytes after treatment with LW6 under normoxia and physioxia

**Fig. 1:**
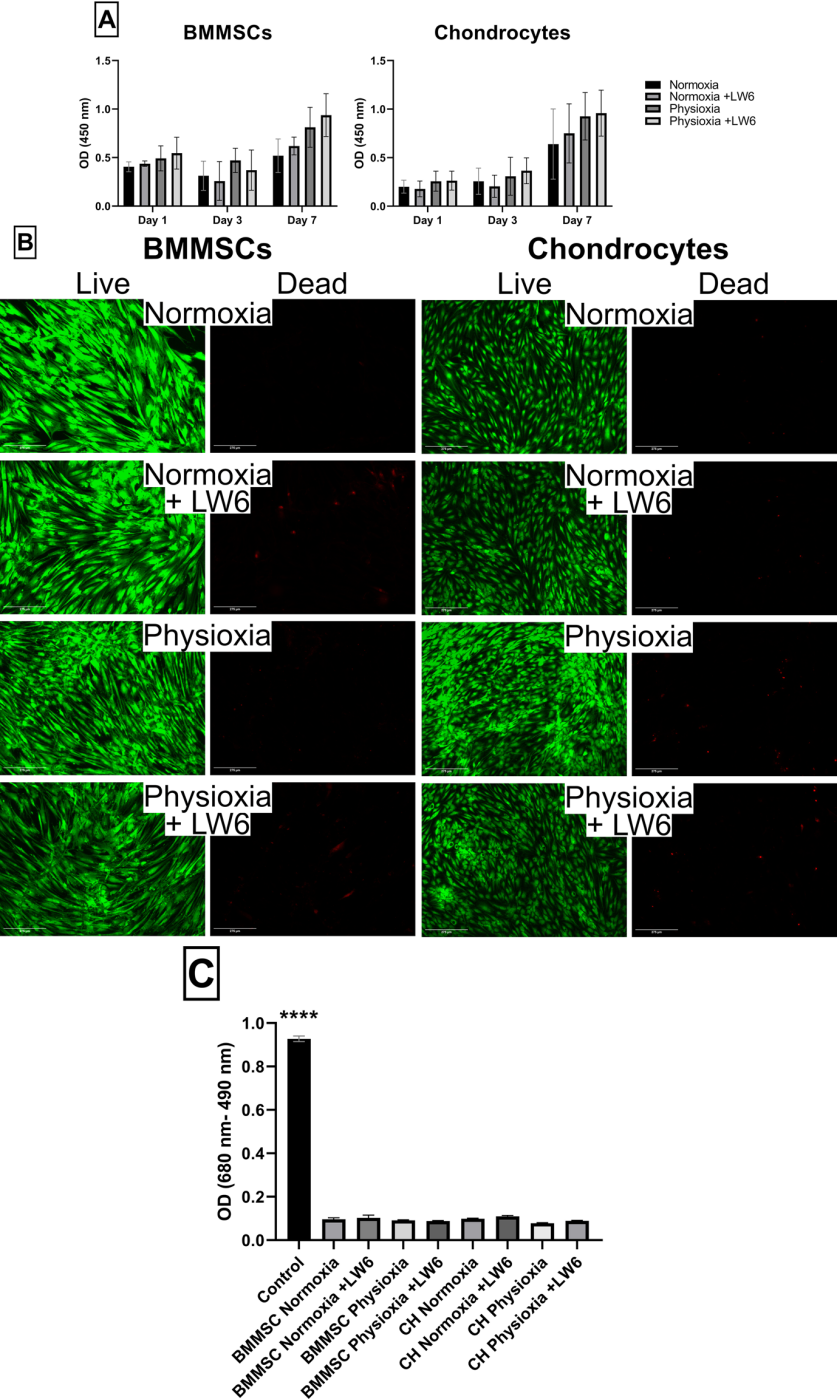
Proliferation and viability of BMMSC and chondrocytes. After cultivation with LW6 under normoxic (21% O_2_) and physioxic (5% O_2_) conditions. **(A)** Proliferation of BMMSCs and chondrocytes after 3 and 7 days (CCK-8 Kit, OD 450 nm). **(B)** Viability of BMMSCs and chondrocytes stained with LIVE/DEAD kit – Calcein-AM (green live cells) and ethidium homodimer-1 (red dead cells). **(C)** Lactate dehydrogenase (LDH) release into BMMSC and chondrocyte (CH) cell medium (with a positive LDH control, measured by OD = 680 –490 nm) after 21 days of cultivation. Data shown with SD from ≥ 3 measurements of each cell type. Statistical significance between the groups was not observed

BMMSC and chondrocyte proliferation/metabolic activity was analyzed using the CCK-8 kit after incubation with LW6 for 3 and 7 days under normoxic and physioxic conditions. The proliferation measurements showed physioxic conditions increased proliferation of BMMSCs and chondrocytes, compared to normoxic conditions during 3 and 7 days in culture (Fig. 1A). LW6 slightly stimulated proliferation of both cell types under normoxia and hypoxia for 7 days (Fig. 1A).

Cellular viability was evaluated using the viability kit LIVE/DEAD under both normoxic and physioxic conditions incubating BMMSCs and chondrocytes with LW6 for 3 and 7 days (Fig. 1B). The cells stained green with LIVE/DEAD kit are alive, while cells stained red exhibit cell death or plasma membrane integrity loss. Data show that neither physioxia nor LW6 did not affect cell viability.

The release of intracellular LDH was chosen to measure a long-term (21 day) effect of physioxia and LW6 on viability of BMMSCs and chondrocytes. The timeframe was chosen to evaluate cellular viability after stimulation with LW6 due to the chondrogenic differentiation protocol, which lasts for 21 days. Release of intracellular lactate dehydrogenase indicates cell death. However, no signs of cell death were observed in cells treated with LW6 neither under physioxia nor normoxia (Fig. 1C). Both cell types remained viable and metabolically active before and after treatment with LW6 under normoxia and physioxia.

### The levels of HIF-1α and its degradation factor VHL in BMMSCs and chondrocytes after treatment with LW6 under normoxia and physioxia

**Fig. 2:**
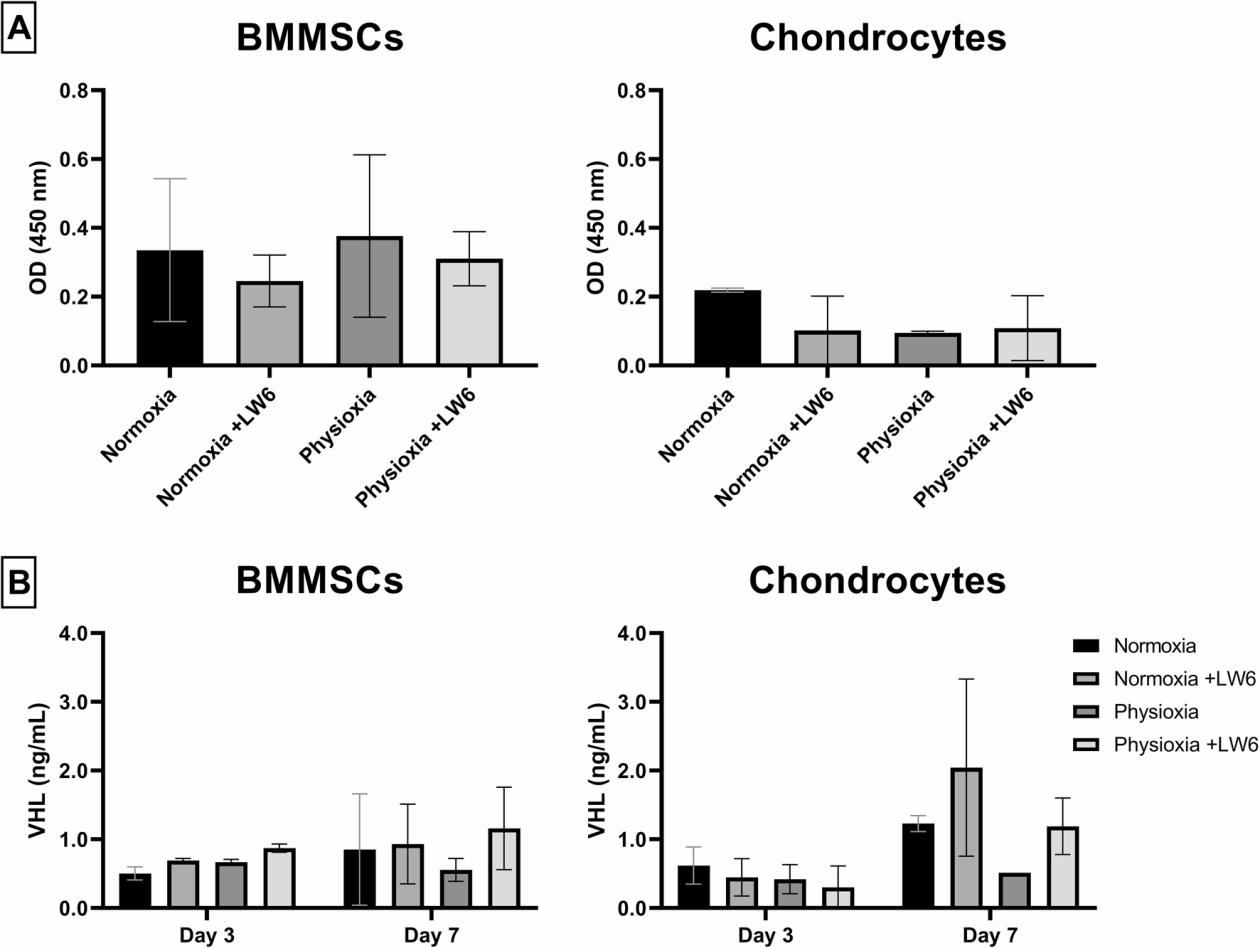
HIF-1α (Hypoxia-inducible factor 1-alpha) and VHL (Von Hippel-Lindau protein) levels in cells after cultivation under normoxic (21% O_2_) and physioxic (5% O_2_) conditions with LW6. **(A)** Relative levels of HIF-1α (OD 450 nm) in BMMSCs and chondrocytes after 3 days (ELISA). **(B)** Concentration of VHL (ng/mL) in BMMSCs and chondrocytes after 3 and 7 days (ELISA). Data shown with SD from ≥ 3 measurements of each cell type. Statistical significance between the groups was not observed

The level of HIF-1α was measured in BMMSCs and chondrocytes after the first 3 days of treatment with LW6. At the control level, BMMSCs had a higher level of HIF-1α compared with chondrocytes (Fig. 2A). HIF-1α levels were slightly upregulated under physioxia in BMMSCs but not in chondrocytes (Fig. 2A). Meanwhile, the HIF-1α inhibitor LW6 tended to suppress HIF-1α in BMMSCs and in chondrocytes during the 3 days of cell growth under both conditions (Fig. 2A).

Since direct HIF-1α measurements did not show significant data, the levels of HIF-1α degradation protein VHL was measured in BMMSCs and chondrocytes under normoxic and physioxic conditions (Fig. 2B). Physioxia did not change the levels of VHL in BMMSCs and chondrocytes after 3 days of cell cultivation, however after 7 days under physioxia, VHL levels in both cell types were suppressed compared to normoxia (Fig. 2B). LW6 increased the levels of VHL in both cell types under normoxia and hypoxia after 7 days of incubation with a more pronounced effect on chondrocytes than BMMSCs (Fig. 2B).

Data suggests that HIF-1α levels in BMMSCs under physioxic conditions do not change during a shorter (3 day) period of incubation compared to chondrocytes. During a longer period (7 days) of incubation under physioxia, HIF-1α degradation tended to reduce in both cell types. LW6 also tends to increase HIF-1α degradation in both cell types under physioxia and hypoxia during a longer period (7 days) of incubation with a stronger effect on chondrocytes than BMMSCs.

### The levels of intracellular calcium ions in BMMSCs and chondrocytes after treatment with LW6 under normoxia and physioxia

**Fig. 3:**
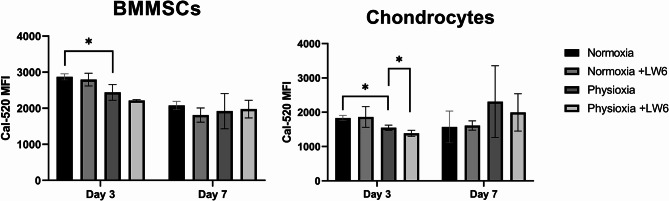
Intracellular calcium levels in BMMSCs and chondrocytes cultivated under normoxic (21% O_2_) and physioxic (5% O_2_) conditions with LW6 (10 µmol). Cells were stained with Cal-520 dye, measured with flow cytometry and the median fluorescence intensity (MFI) was evaluated from ≥ 3 measurements of three cell cultures of each type (*p-value < 0.05). Data are significant comparing normoxia vs. physioxia with/without LW6

BMMSCs and chondrocytes were cultivated for 3 and 7 days with LW6 under normoxia and physioxia for intracellular calcium level analysis (Fig. 3). Calcium levels significantly decreased in both cell types under physioxia during the first 3 days of incubation (*p*-value < 0.05). However, after 7 days, physioxia no longer reduced intracellular calcium level in BMMSCs and even showed a tendency to increase it in chondrocytes.

LW6 also decreased calcium levels in BMMSCs and chondrocytes during the first 3 days of incubation (Fig. 3). However, after 7 days, LW6 had no effect on the intracellular calcium level of BMMSCs, while it slightly reduced it under physioxic conditions in chondrocytes.

These results show that BMMSCs have a higher base intracellular calcium level than chondrocytes, and that physioxia tends to reduce these levels in both cell types during the first 3 days of incubation. A longer incubation period (7 days) with LW6 under either normoxia or physioxia does not affect intracellular calcium in BMMSCs, whereas in chondrocytes it tends to increase under physioxic conditions.

### Mitochondrial respiration and glycolysis measurements in BMMSCs and chondrocytes after treatment with LW6 under normoxia and physioxia

Mitochondrial stress and glycolysis tests were performed to estimate the different bioenergetic states of BMMScs and chondrocytes by monitoring the oxygen consumption rates (OCR) and extracellular acidification rate (ECAR) in live cells after 3 days of incubation.

**Fig. 4:**
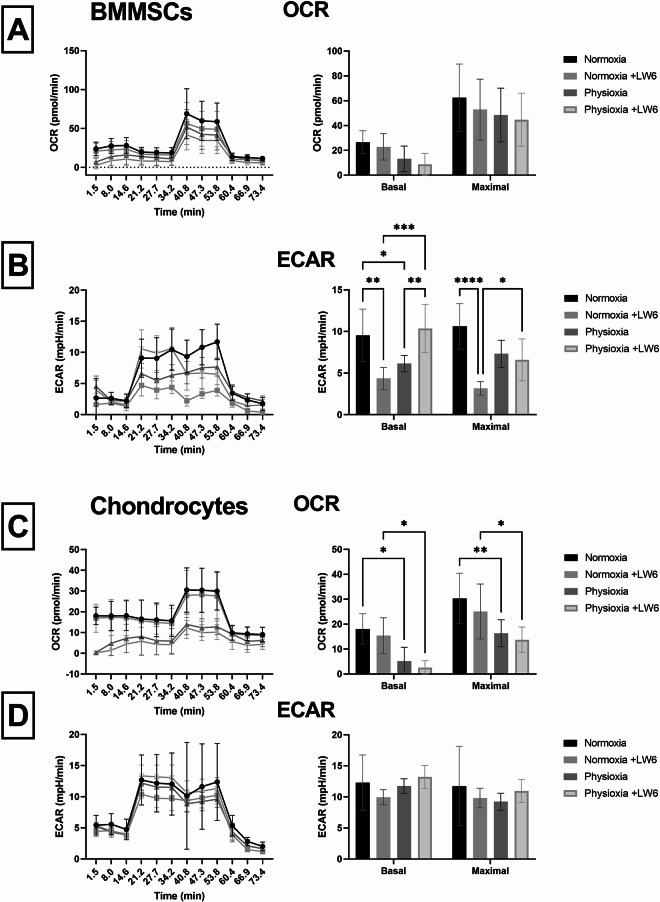
Mitochondrial respiration and glycolysis of BMMSCs and chondrocytes cultivated under normoxic (21% O_2_) and physioxic (5% O_2_) conditions with LW6 (10 µmol). **(A)** BMMSC mitochondrial respiration graph, basal and maximal OCR (Oxygen consumption rate, pmol/min). **(B)** BMMSC glycolytic respiration graph, basal and maximal ECAR (Extracellular acidification rate, mpH/min). **(C)** Chondrocyte mitochondrial respiration graph, basal and maximal OCR (Oxygen consumption rate, pmol/min). **(D)** Chondrocyte glycolytic respiration graph, basal and maximal ECAR (Extracellular acidification rate, mpH/min). Data shown with SD from ≥ 3 measurements of each cell type (*p-value < 0.05; **p-value < 0.01; ***p-value < 0.001; ****p-value < 0.0001). Data are significant comparing normoxia vs. physioxia with/without LW6

Data shows that both cell types had a similar basal respiratory level, while the maximal respiratory capacity was almost twice higher in BMMSCs compared to chondrocytes (Fig. 4A and C). Physioxic conditions decreased basal and maximal OCR in both cell types with the more prominent effect on chondrocytes than on BMMSCs. In addition, LW6 also reduced both basal and maximal OCR mitochondrial respiratory capacity in BMMSCs and chondrocytes under both normoxic and hypoxic conditions with a more prominent effect on chondrocytes than BMMSCs (Fig. 4A and C). Results suggest that physioxia and LW6 act synergistically and suppress the mitochondrial activity in both cell types.

Glycolytic capacity was evaluated by measuring the extracellular acidification rate (ECAR). Data shows that the basal and maximal glycolytic capacity was slightly lower in BMMSCs than in chondrocytes (Fig. 4B and D). Glycolytic activity in BMMSCs was more suppressed by the physioxic (5% O_2_) conditions than in chondrocytes after the first 3 days of incubation. HIF-1α inhibitor LW6 significantly reduced basal and maximal glycolysis in BMMSCs under normoxic conditions. However, under physioxic conditions, LW6 had a slightly stimulating effect on basal and maximal glycolysis in chondrocytes and on basal glycolysis in BMMSCs (Fig. 4B and D). Physioxia and LW6 had a much stronger effect on basal and maximal glycolysis in BMMSCs than chondrocytes under both conditions.

Overall, OCR and ECAR measurements show that physioxia and the HIF-1α inhibitor LW6 have synergistic effects, with stronger impacts on OCR in chondrocytes and on ECAR in BMMSCs. The data also suggests that physioxia and LW6 may preferentially suppress the metabolic pathways that are not naturally dominant in each cell type.

### MDH2 activity in BMMSCs and chondrocytes after treatment with LW6 under normoxia and physioxia

**Fig. 5:**
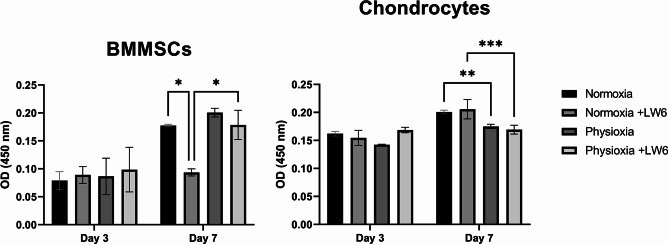
Measurement of malate dehydrogenase 2 (MDH2) activity in BMMSCs and chondrocytes, cultivated for 3 and 7 days under normoxic (21% O_2_) and physioxic (5% O_2_) conditions with HIF-1α inhibitor LW6 (10 µmol). OD was measured at 450 nm. Data shown with SD from ≥ 3 measurements of each cell type (*p-value < 0.05; **p-value < 0.01; *** *p*-value < 0.001). Data was significant comparing normoxia vs. physioxia with/without LW6

The MDH2 activity assay showed that BMMSCs had nearly three-fold lower MDH2 levels than chondrocytes after 3 days of cultivation, but by day 7 these levels had increased to match those of chondrocytes (Fig. 5). Physioxia did not reduce MDH2 levels in BMMSCs at either 3 or 7 days of incubation. In contrast, in chondrocytes the activity of MDH2 was significantly reduced after 7 days of incubation under physioxia (Fig. 5).

After 7 days, LW6 significantly suppressed MDH2 activity in BMMSCs under normoxia, but had no significant effect under physioxia. In chondrocytes, LW6 did not affect MDH2 activity under either growth condition (Fig. 5).

The results suggest that physioxia had a stronger reducing effect on MDH2 activity in chondrocytes than in BMMSCs, whereas LW6 produced a greater reduction in MDH2 in BMMSCs than in chondrocytes after 7 days of incubation. This indicates that physioxia, as a weaker stimulus compared to LW6, was sufficient to reduce MDH2 in chondrocytes but not in BMMSCs, which required the modulation of LW6.

### Chondrogenic differentiation of BMMSCs and chondrocytes in cell sheets after treatment with LW6 under normoxia and physioxia

**Fig. 6:**
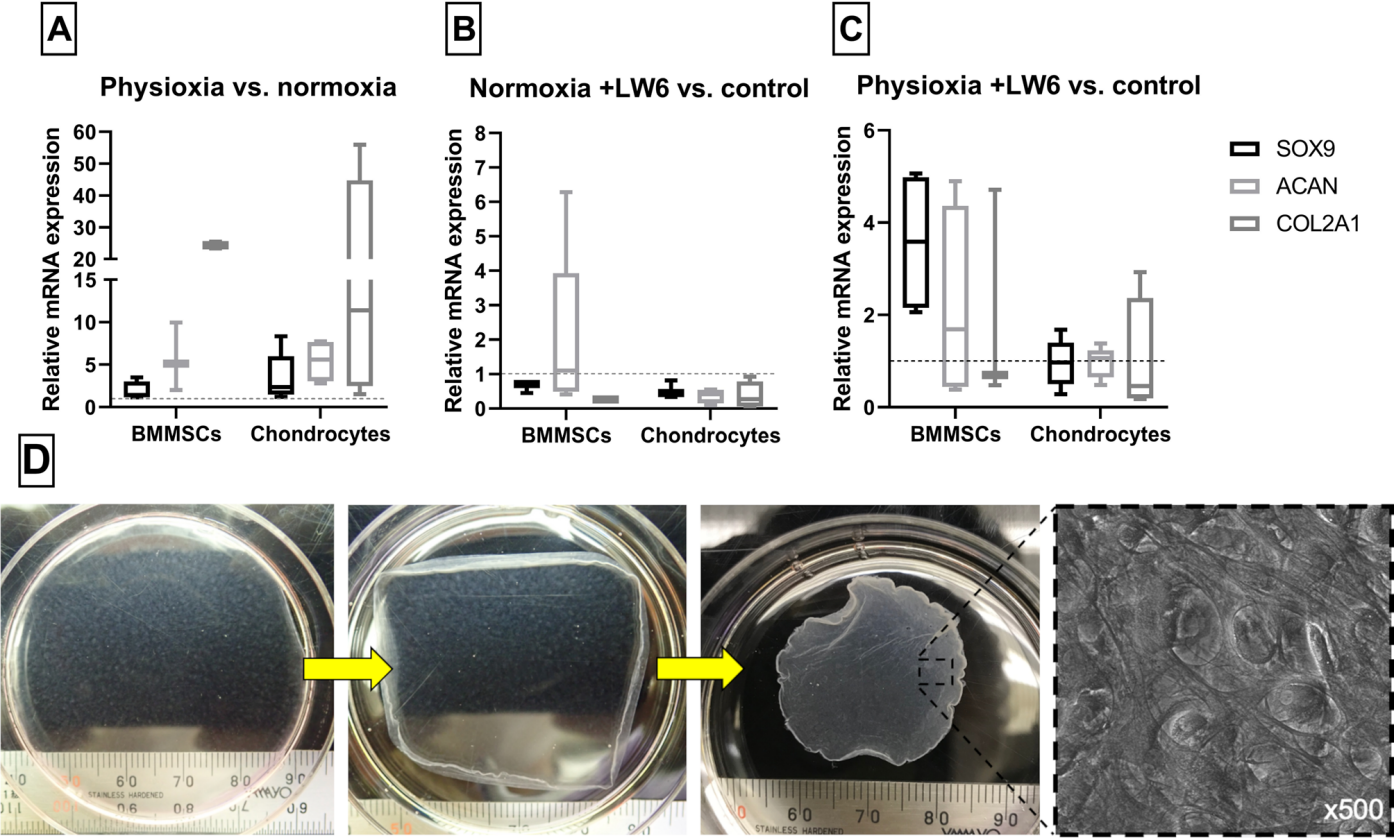
Chondrogenic differentiation of BMMSCs and chondrocytes in cell sheets and gene expression (*SOX9*, *ACAN*, *COL2A1*) analysis in normoxic (21% O_2_) and physioxic (5% O_2_) conditions with LW6 (10 µmol) after 21 days. **(A)** Relative mRNA expression under physioxic conditions (ratio of physioxia/normoxia). **(B)** Relative mRNA expression with LW6 in normoxic conditions (ratio of + LW6/control in normoxia). **(C)** Relative mRNA expression with LW6 in physioxic conditions (ratio of + LW6/control in physioxia). **(D)** Photos and micrograph of a chondrogenic cell sheet in culture, its detachment and extracellular matrix. Data shown with SD from ≥ 3 measurements of each cell type. Statistical differences were calculated as (1) normoxia vs. physioxia; (2) normoxia with LW6 vs. control; (3) physioxia with LW6 vs. control

After 21 days of differentiation and the generation of chondrogenic cell sheets (Fig. 6D), gene expression analysis of chondrogenesis regulating genes (*SOX9*,* ACAN*,* COL2A1*) was performed in BMMSC and chondrocyte cell sheets.

Data indicates that physioxic conditions increased the expression of *SOX9*,* ACAN*,* COL2A1* in both BMMSC and chondrocyte cell sheets, with a more noticeable effect on chondrocytes (Fig. 6A). Under normoxia, LW6 mainly had a suppressing effect on the expression of *SOX9* and *COL2A1* in BMMSCs, whereas in chondrocytes, all three genes (*SOX9*,* ACAN*,* COL2A1*) had their expression downregulated (Fig. 6B). Under physioxic conditions, LW6 had a stronger suppressive effect on *SOX9*, *ACAN* and *COL2A1* expression in chondrocytes than in BMMSCs, though notably reducing *COL2A1* levels in both cell types to almost the same extent (Fig. 6C).

These findings show that physioxia positively affects chondrogenic related genes in BMMSCs and chondrocytes formed in cell sheets. Additionally, data demonstrates the suppressive effect of LW6 on chondrogenic genes, which was more notable in chondrocytes than BMMSCs.

### Biointegration of cell sheets with cartilage explants under normoxia and physioxia

**Fig. 7:**
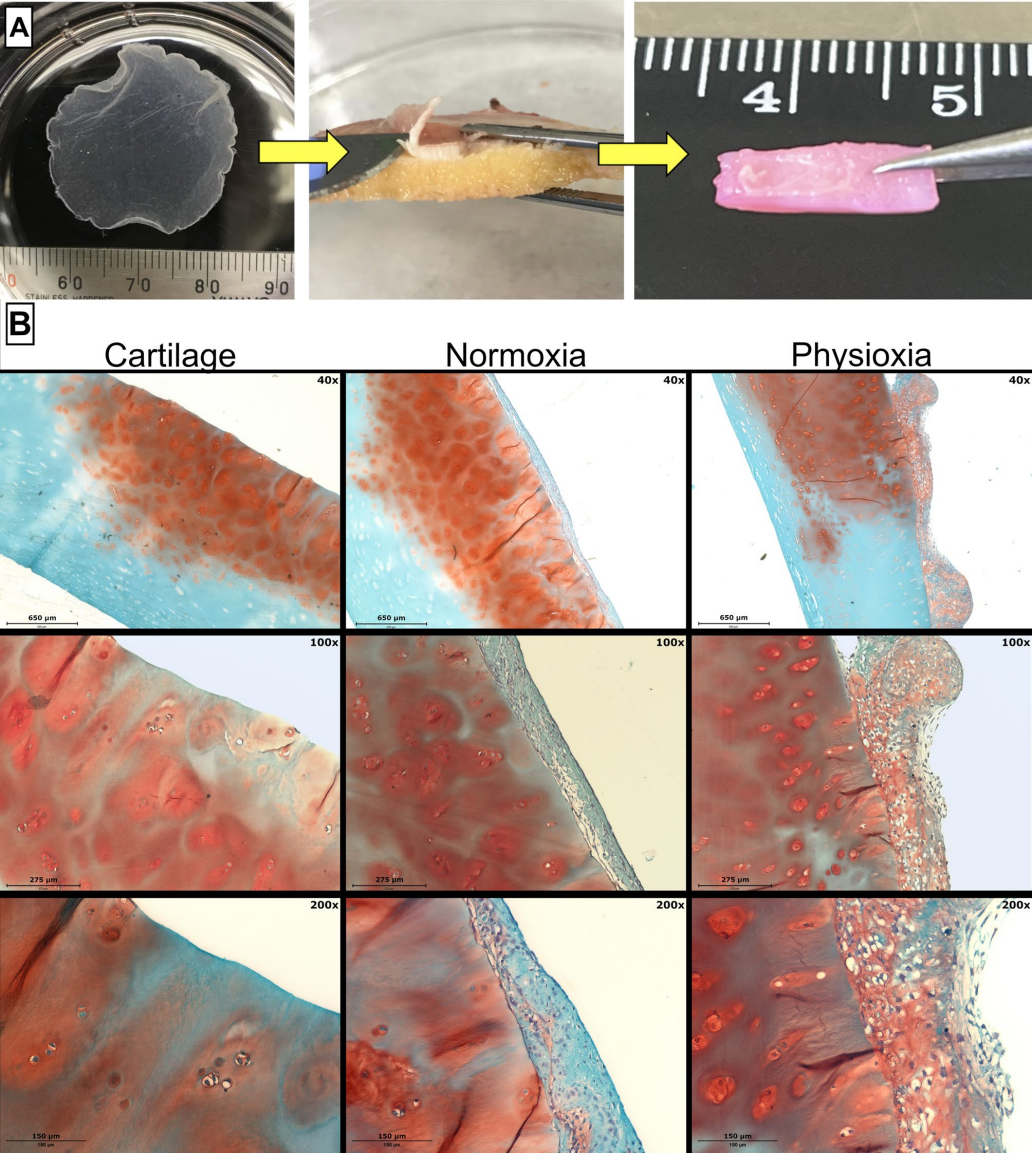
Biointegration of chondrogenic cell sheets onto human articular cartilage explants. **(A)** Chondrogenic cell sheets were added to cartilage explants harvested from osteochondral samples and incubated for 7 days under normoxic (21% O_2_) and physioxic (5% O_2_) conditions. **(B)** Histological Safranin-O staining and microscopy images of cartilage explant integration with chondrogenic cell sheets after 7 days of incubation under different oxygen conditions

Finally, it was investigated how cell sheets integrate with cartilage explants under different oxygen conditions and change their structural composition. Histological staining with Safranin-O and microscopy images revealed that cell sheets better integrated into human cartilage explants under physioxic conditions compared to normoxia, with generation of thicker ECM and higher glycosaminoglycan (GAG) content, more similar to the native cartilage explant (Fig. 7, showing representative data with chondrogenic cell sheet and cartilage explants).

## Discussion

In the present study we aimed to evaluate intracellular changes and the chondrogenic potential of human BMMSCs and chondrocytes in chondrogenic cell sheets under physioxic (5% O_2_) and normoxic (21% O_2_) conditions, treating them with the HIF-1α inhibitor LW6.

LW6, a novel and potential drug for targeting cancerous cells that develop hypoxic conditions, was shown to effectively attenuate cancer formation by promoting cancer cell apoptosis via indirect HIF-1 inhibition. LW6 was shown to induce apoptosis of A549 lung cancer cells under 1% of hypoxia by depolarizing mitochondria and inhibiting the accumulation of HIF-1 [11]. This pharmacological strategy could be a potential way of preventing cancerous tumor formation, however, if considered a potential anti-cancer therapeutic for future clinical studies, it is crucial to analyze its possible mechanisms in other HIF-dependent tissues, including cartilage.

The early formation of articular cartilage – chondrogenesis, is highly sensitive to oxygen changes and HIFs play an important role in influencing the process. BMMSCs originally form cartilage during fetal development and are considered as one of the main stem cell types for cartilage regeneration during the progression of OA, or after trauma [17, 18]. Therefore, it is important to understand if HIF-1 inhibitors, such as LW6, can affect the chondrogenic differentiation potential of BMMSCs in vitro. Physioxia plays a key role in mature chondrocyte homeostasis, where the upper layer of cartilage is subjected to physioxia of around 5% O_2_ [19]. For the application of cell sheet technology on the upper layers of cartilage, we applied and maintained 5% physioxic conditions throughout our experiments in comparison with standard normoxic conditions (21% O_2_), which are generally used for MSC culture and differentiation in laboratories, with a growing consensus on the negative effects and modulations normoxia can potentially have on MSC and other stem cell metabolic capacities, differentiation potential and application for tissue engineering purposes [20].

Firstly, we analyzed the proliferation/metabolic activity and viability of both cell types – BMMSCs and chondrocytes – after 3, 7 and 21 days of incubation with LW6 under physioxic and normoxic conditions. Data showed that LW6 did not affect cellular proliferation/metabolic activity, however physioxic conditions increased proliferation/metabolic activity in BMMSCs and tended to increase metabolic activity in chondrocytes after 7 days in culture. We also observed that after 21 days in culture with or without treatment with LW6, both cell types remained viable under normoxia and physioxia.

The benefits of physioxia in MSCs and chondrocytes has been established in previous studies – as bone marrow and cartilage have a gradient oxygen concentration of between 1 and 7% O_2_ and 1–7% O_2_ respectively [21, 22], hypoxia can increase cell viability, promote proliferation, help maintain MSC stemness and multipotent properties through the expression of certain stemness factors (such as Oct-4, Sox2, Nanog) [21, 23, 24]. Increased chondrocyte proliferation in physioxia can be explained by the natural physiological conditions in cartilage, which are favorable to maintain the chondrogenic phenotype, promote chondrocyte proliferation, growth and metabolism, all of which has a beneficial effect on chondrogenesis and cartilage homeostasis [9, 25].

The molecular mechanisms of LW6 work through the indirect inhibition of HIF-1α, which was shown to be more stable in BMMSCs compared to chondrocytes after 3 days of incubation. LW6 increased the VHL protein level, especially after 7 days of incubation, suggesting that HIF-1α degradation was elevated under LW6 treatment. VHL functions as a master regulator of HIF activity by binding to hydroxylated HIF-1α subunits for ubiquitylation and rapid proteasomal degradation under normoxic conditions. Therefore, an increase of VHL is associated with a higher intensity of HIF-1 suppression/degradation [26]. The findings of this study confirm the mechanism of LW6 action previously reported in colon cancer cells, where inhibitor-induced upregulation of VHL was shown to promote cancer cell death [12].

Thereafter, we analyzed intracellular calcium ion changes in both cell types under different oxygen conditions. It is known that calcium plays a major role in many of the cellular signaling pathways [27], among them an important role in proliferation and differentiation of MSCs [28]. In this study, the disruption of calcium ion levels in BMMSCs and chondrocytes was observed after the short-term (3 days) incubation of cells in physioxic conditions, with the addition of LW6 also lowering calcium ion levels in chondrocytes. Changes in calcium ion concentration and calcium channel activity can be triggered by various stimuli, including drugs, mechanical stress, physical factors, and electrical stimulation [29, 30].

Furthermore, we have evaluated the activity of mitochondrial respiration and glycolysis in both cell types under normoxic and physioxic conditions. The typical metabolic state of undifferentiated MSCs is mainly glycolytic, with an elevated glycolytic flux being associated with cell stemness [31], while differentiation into various cell types may shift them from glycolysis to oxidative phosphorylation [32, 33]. Cells possessing stemness properties typically exhibit lower levels of ROS and antioxidant enzymes, as well as reduced levels of mitochondrial and ROS-generating proteins due to their lower mitochondrial activity [34]. Due to the avascular and metabolically slow environment of cartilage resulting in a lack of available oxygen, the predominant way to produce ATP in chondrocytes is mainly glycolytic [24]. Chondrocytes are naturally adapted to a low oxygen environment, therefore in order to mimic the in vivo environment, physioxic conditions should be an important factor to apply and consider when testing cellular responses and functions to different drugs.

Data of this study showed that BMMSCs and chondrocytes were generating energy via both mitochondrial respiration and glycolysis under normoxic conditions, though with an observable difference in capacity – BMMSCs had a higher basal and maximal oxygen consumption rate compared to chondrocytes, while chondrocytes exhibited a higher rate of glycolysis, which was more stable and resistant to any changes caused by oxygen and LW6. Physioxia significantly decreased the electron transport chain activity via lowering OCR after 3 days of culture. With the addition of LW6, the decrease of OCR in both cell types was even more pronounced, which coincides with previous research showing that LW6 lowered the oxygen consumption rate in cancer cells [13]. Glycolysis was not restored in BMMSCs after 3 days of incubation under physioxia compared to normoxia, however, LW6 tended to promote a significantly higher basal glycolysis in physioxic conditions – possibly related to its inhibitory effects on MDH2 and the TCA cycle. These results reveal distinct metabolic profiles in BMMSCs and chondrocytes: chondrocytes are more sensitive to changes in mitochondrial respiration, whereas BMMSCs respond more strongly to glycolytic modulation induced by oxygen levels and LW6.

Another factor involved in LW6 action is MDH2 activity. MDH2 is a mitochondrial enzyme, utilizing the NAD/NADH system to convert malate/NAD + into oxaloacetate/NADH during the tricarboxylic acid cycle (TCA cycle) [15], therefore, its inhibition is directly related to mitochondrial metabolic changes in the cells. In our study, data showed MDH2 activity was higher in chondrocytes compared to BMMSCs at normoxic conditions. MDH2 activity was significantly lowered by physioxia in chondrocytes after 7 days of incubation. LW6 had a strong suppressing effect on MDH2 in BMMSCs under normoxia, and a weaker suppressive tendency in physioxia, while it did not have any significant effect in chondrocytes after 7 days of incubation. These results again highlight metabolic differences between BMMSCs and chondrocytes: LW6 inhibited MDH2 only in BMMSCs, whereas in chondrocytes MDH2 level was influenced by physioxia but not by LW6. Further experiments into these mechanisms could elucidate results.

Finally, chondrogenic capacity of BMMSCs and chondrocytes were evaluated in a cell sheet model under both normoxic and physioxic conditions and stimulation with LW6. Cell sheet technology is a scaffold-free method used in tissue engineering based on culturing cells on a thermoreactive polymer which can exhibit hydrophobic or hydrophilic properties depending on the temperature. Under standard cell culture incubating conditions (37 °C), the cells adhere to the hydrophobic polymer, while lowering the temperature (around 20 °C) the detachment of cell layers with a fully intact extracellular matrix (ECM), surface/ECM proteins and cell junctions occurs, which can be vital for the effective cell biointegration and long-term post-transplantational survival in vivo [35].

**Fig. 8:**
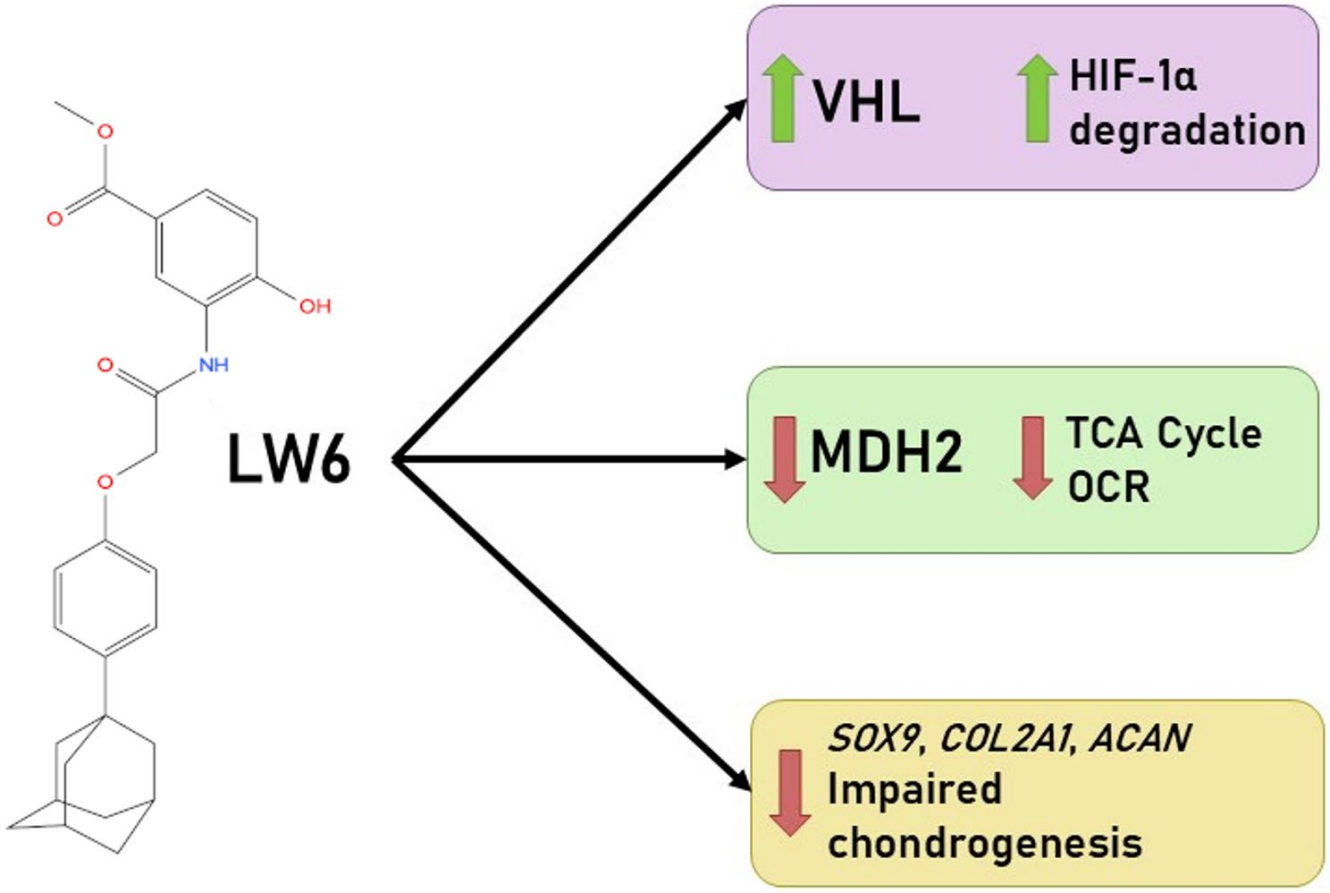
The suggested mechanism of LW6 action on the chondrogenic potential of BMMSCs and chondrocytes in cell sheets. VHL - Von Hippel-Lindau protein; HIF-1α - Hypoxia-inducible factor 1α; MDH2 - malate dehydrogenase 2; TCA - tricarboxylic acid; OCR - oxygen consumption rate; *SOX9* - SRY-box transcription factor 9 gene; *COL2A1* - collagen, type II, alpha 1 gene; *ACAN* - aggrecan gene

The data of this study demonstrate that physioxic conditions upregulate the expression of chondrogenic genes (*SOX9*, *ACAN*, *COL2A1*) in both cell types cultivated in cell sheets, while LW6 downregulates them with the exception of BMMSCs under physioxia. These results are novel and LW6’s effect on chondrogenic potential of BMMSCs and chondrocytes have not been described before. However, the effect of other HIF-1α modulators has been studied in a similar manner, downregulating the *SOX9*, *ACAN* and *COL2A1* genes in rat and human chondrocytes [36, 37]. The summarised mechanism of LW6 action, based on this study findings, is presented in Fig. 8.

We also compared how different physiological conditions might influence the biointegration of cell sheet layers onto cartilage tissue explants. Thorp et al., 2020 conducted a similar study [38], where they analyzed cell sheet application for cartilage under hypoxic conditions and compared them to 2D sheets and pellets. The study demonstrated that the 3D sheets produce more cartilage-related genes (*COL2A1*,* ACAN*,* SOX9*) as compared to 2D control sheets (without chondrogenic induction), and possess similar differentiation properties as cell pellets. They also showed successful physical adhesion of cell sheets to cartilage explants, with increased expression of adhesion molecules between the two surfaces [38]. Stacking these cell sheet constructs on top of each other before application can also increase the expression of chondrogenic genes, described in their later studies [39]. Similar to Thorp et al., we confirm the better integration of cell sheet into human cartilage explants under physioxic conditions compared to normoxia, with generation of thicker ECM and higher glycosaminoglycan (GAG) content similar to the native cartilage. Data revealed cell sheets being suitable for the cartilage tissue regeneration under the native physioxic cartilage conditions.

These findings have direct translational relevance for cartilage repair strategies. By demonstrating that physioxia supports metabolic activity, calcium homeostasis, and chondrogenic differentiation, our results highlight the importance of recreating low-oxygen conditions in MSC-based therapies and engineered constructs for OA treatment. Conversely, the inhibitory effects of LW6 highlight a potential risk when HIF-1α/MDH2 inhibitors are administered systemically in patients who may also require cartilage regeneration or have ongoing joint degeneration. Understanding these interactions may guide safer therapeutic planning, optimize microenvironmental conditions for cartilage repair, and inform the development of regenerative approaches that preserve HIF-dependent pathways essential for tissue homeostasis.

### Study limitation and future directions

This study has several limitations that should be addressed in future work. First, the sample size was relatively small, and inclusion of a larger number of donors will be important to increase statistical relevance and improve the generalizability of the findings. Second, although we examined several key upstream pathways involved in HIF-1α regulation, a comprehensive systems-level analysis (e.g., transcriptomics, proteomics, or phospho-signaling profiles) is still needed to fully map the molecular cascade through which LW6 interferes with cartilage formation under physioxic versus normoxic conditions.

In addition, in the present study we used only female donors, which may introduce sex-related bias, given known metabolic and cartilage differences. Future sex-comparative studies could clarify differential MSC/chondrocyte responses to HIF-1 modulation under physioxia, and strengthen understanding of personalized cartilage engineering.

Adult articular cartilage and MSC-based repair strategies heavily rely on the interaction of HIF-1α signaling, matrix production and survival in low-oxygen environments. Thus, systemic use of anti-cancer LW6 may pose risks for patients with OA, joint injuries, or those undergoing cartilage-repair procedures. Further in vivo and longitudinal studies are required to determine clinically relevant exposure thresholds, tissue-specific sensitivities, and strategies to mitigate unwanted effects on musculoskeletal tissues while preserving the anti-tumor benefits of LW6.

## Conclusions

This study demonstrates that physioxia enhances BMMSC and chondrocyte metabolism, promotes chondrogenic gene expression, and improves the structural integrity and biointegration of scaffold-free cell sheets into human cartilage explants. LW6, in contrast, disrupts several HIF-1α–dependent pathways: suppresses MDH2 activity, alters intracellular calcium signaling, and reduces expression of chondrogenic markers, indicating potential off-target inhibitory effects on cartilage-forming cells. These results highlight the importance of physioxic conditions for cartilage engineering studies and suggest that anti-cancer HIF-1α inhibitors, such as LW6, should be used with caution in contexts where cartilage maintenance or regeneration is clinically relevant.

## Data Availability

The datasets used and/or analysed during the current study are available from the corresponding author on reasonable request.

## Abbreviations

HIF-1: Hypoxia-inducible factor 1
HIF-1α: Hypoxia-inducible factor 1-alpha
OA: Osteoarthritis
MSCs: Mesenchymal stem cells
BMMSCs: Bone marrow Mesenchymal stem cells
CH: Chondrocytes
ECM: Extracellular matrix
LW6: 3-[2-(4-Adamantan-1-yl-phenoxy)-acetylamino]-4-hydroxy-benzoic acid methyl ester
VHL: Von Hippel-Lindau protein
MDH2: Malate dehydrogenase 2
LDH: Lactate dehydrogenase
OCR: Oxygen consumption rate
ECAR: Extracellular acidification rate
